# Status of Palliative Oncology Care for Children and Young People in Sub-Saharan Africa: A Perspective Paper on Priorities for New Frontiers

**DOI:** 10.1200/GO.21.00102

**Published:** 2021-09-21

**Authors:** Eve Namisango, Nickhill Bhakta, Joanne Wolfe, Michael J. McNeil, Richard A. Powell, Solomon Kibudde, Emmanuel B. K. Luyirika, Vivienne Mulema, Chris Feudtner, Justin N. Baker

**Affiliations:** ^1^ African Palliative Care Association, Kampala, Uganda; ^2^ Cicely Saunders Institute, King's College London, London, United Kingdom; ^3^ Global Pediatric Medicine, St Jude Children's Research Hospital, Memphis, TN; ^4^ Department of Pediatrics, Boston Children's Hospital, Boston, MA; ^5^ Department of Psychosocial Oncology and Palliative Care, Dana-Farber Cancer Institute, Boston, MA; ^6^ Division of Quality of Life and Palliative Care, Department of Oncology, St Jude Children's Research Hospital, Memphis, TN; ^7^ Department of Primary Care and Public Health, Imperial College London, London, United Kingdom; ^8^ MWAPO Health Development Group, Nairobi, Kenya; ^9^ Department of Medical Oncology, Uganda Cancer Institute, Kampala, Uganda; ^10^ African Palliative Care Association, Kampala, Uganda; ^11^ Clinton Health Access Initiative, Kampala, Uganda; ^12^ Department of Medical Ethics, The Children's Hospital of Philadelphia, Philadelphia, PA; ^13^ Departments of Pediatrics and of Medical Ethics and Health Policy, The Perelman School of Medicine of the University of Pennsylvania, Philadelphia, PA; ^14^ Division of Quality of Life and Palliative Care, Department of Oncology, St Jude Children's Research Hospital, Memphis, TN

## Abstract

The burden of cancer disproportionately affects low- and middle-income countries. Low 5-year survival figures for children with cancer in low-income countries are due to late presentation at diagnosis, treatment abandonment, absence of sophisticated multidisciplinary care, and lack of adequate resources. The reasons for late presentation are partly due to limited awareness of cancer symptoms, high treatment costs, and facility-level barriers to timely access to treatment. Given the systemic challenges, the regional need for palliative oncology care for children care is high. Despite the enormity of the need for palliative oncology for children with cancer in Africa, its level of development remains poor. This paper presents the evidence on the status of palliative oncology care for children in sub-Saharan Africa.

**METHODS:**

This review provides an overview of the current status of palliative oncology care for children in sub-Saharan Africa, using the WHO building blocks for health systems strengthening as reference points, before proposing a forward-looking prioritized agenda for its development.

**RESULTS:**

We noted that survival rates for children with cancer remain much poorer in Africa compared with developed countries and palliative oncology care resources are scant. Our results also show low coverage for palliative oncology care services for children, lack of a critical mass of health workers with the skills to deliver the care, a lack of robust documentation of the burden of cancer, widespread lack of access to essential controlled medicines, limited funding from government and limited coverage for palliative oncology care in most cancer control plans.

**CONCLUSION:**

This review highlights priority areas for action that align to the WHO health system building blocks for strengthening health systems.

## INTRODUCTION

Epidemiologic data on childhood cancer survival are important for policy development, priority setting, and planning. As per global estimates of diagnosed cases, the 5-year net childhood survival is 37.4%; however, there are large regional variations ranging from 8.1% (4.4-13.7) in eastern Africa to 83.0% (81.6-84.4) in North America.^1^ Differences in survival gaps between developing and developed countries can be as high as 70% and can be explained by gross inequities in treatment access, quality of care, and its affordability.^1^ Other explanatory factors include late presentation at diagnosis, treatment abandonment, absence of sophisticated multidisciplinary care, and lack of adequate resources.^1^ Reasons for late presentation include limited awareness of cancer symptoms, high treatment costs, and facility-level barriers for timely access to treatment.^2^

**CONTEXT**

**Key Objective**
To establish the state of palliative care in pediatric oncology services in sub-Saharan Africa.
**Knowledge Generated**
Palliative care in pediatric oncology in sub-Saharan Africa remains largely underdeveloped. A public health systems approach prioritizing service delivery, health workforce, information systems, access to essential medicines, health systems financing, leadership, and governance should be prioritized for service development.
**Relevance**
Promoting equitable access to palliative care in pediatric oncology in sub-Saharan Africa can only be achieved via the public health model. We propose health systems–based strategies to support the integration of palliative care in existing pediatric oncology services.

The incidence of childhood cancer ranges from 50 to 200 per million children and 90 to 300 per million adolescents.^3^ These data, however, likely substantially underestimate the true incidence rates of pediatric cancer in Africa, where widespread lack of robust childhood cancer registries makes it problematic to collect surveillance data^4^ and existing data are often incomplete and provide very limited information on multiple childhood cancers.^5^ Estimates from African countries with functioning registries show an increasing incidence of pediatric cancers, for example, a survey that included 21 centers from 18 sub-Saharan African countries. For example, in a survey that included 21 centers from 18 sub-Saharan African countries, with data that differed from center to center, including cases from 1985 to 2011, the proportion of childhood cancer of all cancers ranged between 1.4% in Ghana and 10.0% in Rwanda. In Southern Africa, Kaposi sarcoma was the most common malignancy in children in Mozambique (15.8% of all cases) and the second most common in Zambia (15.6%) and in Malawi (12.4%). In Eastern Africa, Uganda recorded Kaposi sarcoma as the most common tumor in children (22.0%), whereas two Kenyan centers reported mainly Burkitt lymphoma (25.1% and 37.1%, respectively). In Central Africa, Congo classified retinoblastoma as the most common childhood cancer with an incidence of 20.1%. In Western Africa, non-Hodgkin lymphoma was the most common in Ghana (53.6%), in Ivory Coast (73.6%), and in Mali (32.7%). Nephroblastoma remains the most common solid tumor in Africa exceeding 10% of total pediatric cancers in many countries (Rwanda 21.3%, Senegal 22%, Ivory Coast 14.5%, Mali 17.6%, and Congo 15.5%).^5^

A primary concern is the lack of data on children whose details are never documented in cancer registries and those possibly misdiagnosed, leading to a substantial underestimate^6^ and missed opportunities for patients to be diagnosed and treated.^7^ Available data are therefore more a reflection of the number of cases of childhood cancers being identified that progress into care for treatment.^7^ These estimates continue to show a two-to-threefold increase in their incidence in low-index compared with high-index Human Development Index countries,^8^ which may be attributed to improved case finding and diagnosis of cancer.

Given the challenges inherent to disease burden and the health system related to children obtaining access and receiving quality childhood cancer care, the regional need for both primary (ie, from pediatric oncologists) and speciality (ie, trained palliative care physicians) palliative care in pediatric oncology is high.^9^ Indeed, the WHO recommends the integration of palliative care into pediatric oncology services to improve the overall quality of care and associated outcomes.^10^ In high-income countries, pediatric palliative care (PPC), underpinned by a person-centered approach, improves care satisfaction and quality of life (QoL).^11^ There is also emerging evidence for the cost-effectiveness of specialist PPC providers and their ability to improve symptom management, mitigate health-related suffering, and improve the QoL of affected children and their families' care in resource-limited settings.^12^ Despite the enormous need for palliative cancer care for children in Africa,^13^ the level of service development remains poor.^14^ The continent is, however, attempting to prioritize the development of palliative care services as a core component of care throughout the continuum of life, in line with the World Health Assembly Resolution.^10^

## FRAMEWORK

Providing an overview of the current status of oncologic PPC in sub-Saharan Africa and the priority needs to improve it is critical to informing person-centered service development that mirrors the needs of patients and their families and optimizes care outcomes.^15^ Our analysis is underpinned by the WHO health system building framework. This framework constitutes each of the following blocks: (1) service delivery, (2) health workforce, (3) health information systems, (4) access to essential medicines, (5) financing, and (6) leadership or governance.^16^ This framework was preferred as it offers a generic approach to building resilient health systems, as well as their monitoring and evaluation, that allows for regional comparison of strengths and gaps in health systems and for setting prioritized service development agenda. Prioritizing these building blocks helps guide a minimum scope to stimulating systems' strengthening, monitoring performance, and evaluation. Each building block is discussed in the sub-Saharan African context, with priorities highlighted to foster service development in line with the World Health Assembly Resolution.^10^

## HEALTH SERVICE DELIVERY

Pediatric cancer care populations have a high burden of complex and multidimensional symptoms^17,18^ that are associated with severe health-related suffering.^9^ Nevertheless, the availability of PPC in the region remains critically limited, with some countries having no known activity or capacity.^14^ This lack of supportive care within cancer health services has been given as a justification for withdrawing active cancer treatment in some situations, thereby negatively affecting treatment outcomes.^19^ Given the importance of multidimensional care in the pediatric cancer service development continuum, the integration of PPC in pediatric oncology supportive care services warrants emphasis^20^ and should be considered as *standard care*^21^ alongside traditional interventions such as transfusion medicine.

Integrated service development is also essential to ensure that children receive high-quality evidence-based services. In a systematic review that established the evidence for PPC models, interventions, and outcomes, the authors noted the lack of outcomes' evidence, among other knowledge gaps.^22^ Similarly, studies have highlighted the lack of appropriate outcome measures in this field,^23^ despite being integral to the quality assessment and health quality improvement framework.^24^ The lack of robust outcome measures means that assessing the effectiveness of services, or developing an evidence base for the impact of interventions, is problematic. Although tremendous progress has been made in developing appropriate outcome measures for adult palliative care,^25^ much more work needs to be undertaken in PPC. Despite the lack of a robust evidence base, few studies conducted provide an insight into the configuration of person-centered care from the perspective of children and their families in sub-Saharan Africa,^26^ the pressing multidimensional needs of children with cancer,^27^ and the potential benefits of person-centered PPC in resource-limited settings.^12^

## HEALTH WORKFORCE

**FIG 1:**
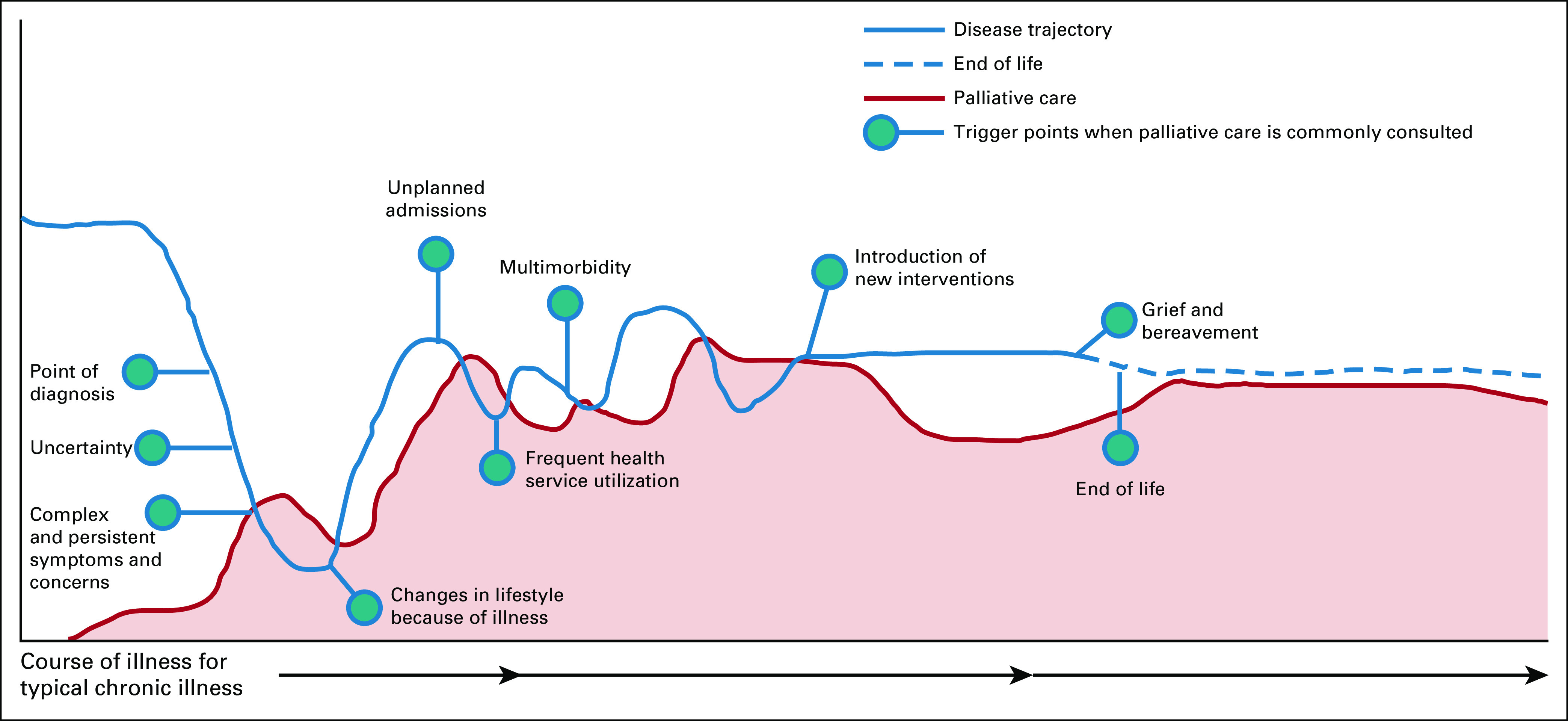
Trigger points for palliative care along the disease trajectory.

Poor cancer survival in sub-Saharan Africa has been partly attributed to poor health care systems, lack of sufficient numbers of health workers, and limited staff education and training opportunities to meet patients' and families' care needs.^28^ Palliative care is of necessity multidisciplinary, as meeting the multidimensional needs of children and their families requires multidisciplinary teams. For example, if there is uncertainty about the outcome of treatment, child and family needs range from participating in difficult conversations (breaking bad news)—which must be sensitively addressed—and managing multidimensional symptoms and concerns, supporting family and sibling needs, and providing advance care planning before a disease's terminal phase and grief and bereavement support, all under a person-centered approach (Fig 1).^18,29^ Palliative care integration is not only important in improving end-of-life outcomes but can also improve outcomes of children living with advanced cancer and their caregivers and families. Quality care matters irrespective of the expected treatment outcome as poor-quality deaths can result in complicated grief and bereavement outcomes, which can, in turn, contribute to poor mental health and significant morbidity in surviving family members.^30^

The number of patients accessing PPC is low (3%), and this is explained by the poor development of PPC services. The estimated services delivering PPC stand at 4% for the AFRO African region.^31^ The 2017 Atlas for Palliative Care in Africa also indicated low availability of PPC services; the country with the highest number of service outlets was South Africa (with 20), followed by Nigeria (10), Malawi (seven), and Zambia (four).^14^

Pediatric oncology service development equally remains low. In 1990, only four pediatric oncology services were available in North Africa; over time, 22 centers have been opened up in 18 Francophone countries.^32^ In a cross-sectional study that aimed to profile the status of resources for pediatric oncology services in a cross-section of hospitals in Africa, it was shown that the care was largely provided by nonpediatric oncologists and the lack of radiotherapy services was notable (available at 55% of the 38 facilities) Broadly speaking, palliation services were reportedly available at 71% of the facilities.^33^

A robust human resource workforce is pivotal to integrated pediatric palliative oncology service development. Cancer care strategies have been mainstreamed to include prevention and early detection, treatment (by surgery, radiation therapy, or chemotherapy), supportive care, palliative care, end-of-life care, and survivorship. Such complex care models demand trained multidisciplinary teams with skills to meet the multidimensional needs of patients and their families.^34^ The status of human resource development for pediatric oncology in sub-Saharan Africa is, however, still in the embryonic stage, and consequently, the importance of capacity building through access to sustainable quality education and training cannot be overemphasized. The regional human resource inventory is characterized by too few pediatric oncologists and specialized nurses and a lack of other multidisciplinary support members (including palliative care providers).

Key players, such as the Africa Pediatric Fellowship Network, WHO technical advisors, Health Ministry funding, and the Education in Palliative and End-of-Life Care pediatrics curriculum initiative,^35^ are training or capacity building platforms. Care providers can be trained online through continuous medical education sessions, fellowships, master's degree trainings, short courses through deployment in palliative care positions, lectures and grand rounds, bedside teaching, weekend courses, and placements in palliative care organizations. In Table 1, we provide a summary of existing courses available in Africa.

**TABLE 1:**
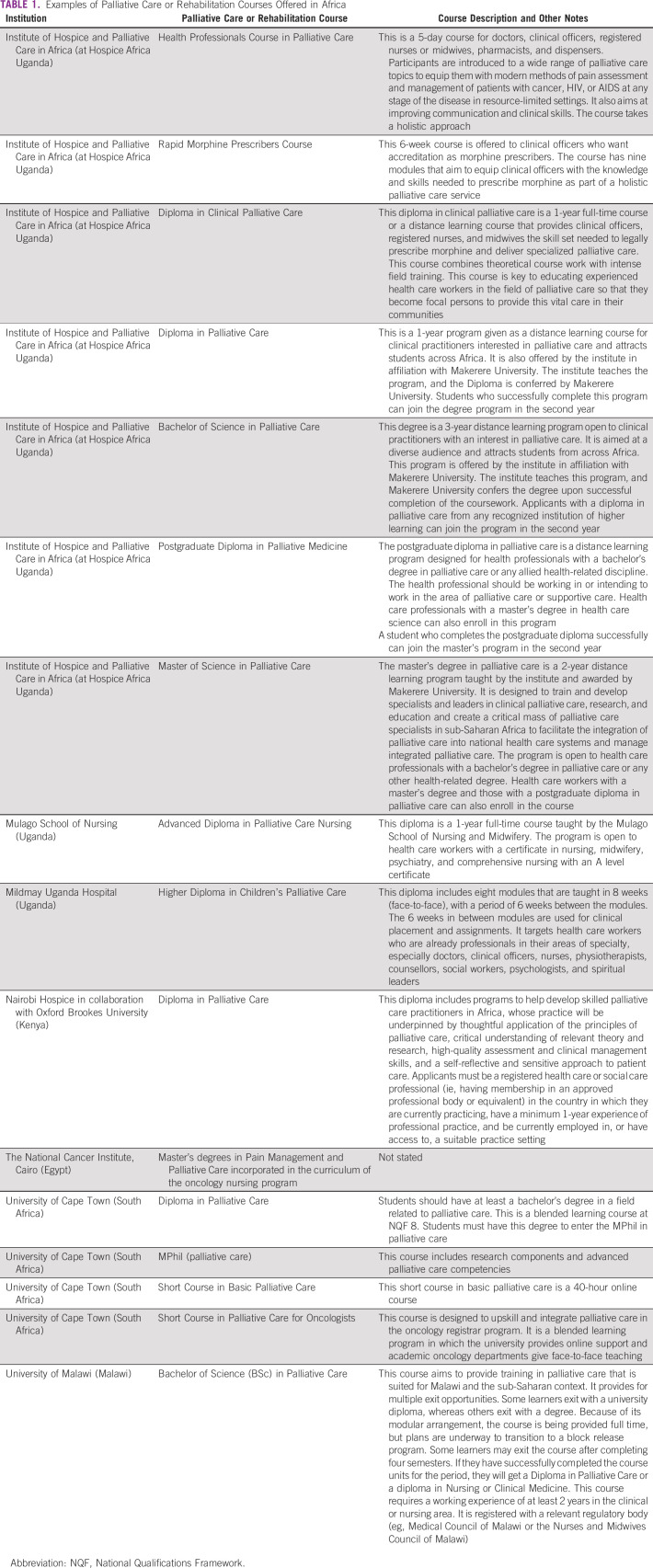
Examples of Palliative Care or Rehabilitation Courses Offered in Africa

Approximately nine countries in Africa have a national palliative care plan or program to strengthen the development of a public health primary care approach to ensure access to this essential service,^36^ and continuous development of education and training opportunities has been recorded in the region,^37^ with curricula integrating PPC. These should serve as an opportunity to strengthen pediatric cancer services by integrating PPC to deliver care that meets the needs of patients and their families. Importantly, such plans are insufficient without adequate budgetary support, a critical consideration.

## HEALTH INFORMATION SYSTEMS

Robust health information systems are pivotal to service development. The lack of quality local data is a barrier to evidence-based practice, data-driven decision making, and the development of services tailored to the needs of African populations.^38^ For example, data on the incidence and prevalence of cancers are largely lacking because of the limited coverage of national cancer registries, which compromises documenting gaps and reporting morbidity and mortality data. In 2012, the African Cancer Registry Network reported that only 22 of the 54 countries in sub-Saharan Africa (40.1%) were contributing to this database, and this only slightly increased to 25 (46.3%) in 2014.^39^ Even where such registries are available, the data quality is largely poor and, as such, the region routinely relies on estimates to understand the extent of the cancer burden.

The registry of cancer cases becomes more complicated with childhood cancers—given their low incidence compared with those for adults—as large populations may be required to generate substantial measures of disease burden.^40^ The lack of data sharing efforts is also a challenge in this field as partners continue to work in silos. For example, the African Cancer Registry Network does not share data publicly, which makes health services research challenging. The increasing concerns over and development of global privacy laws exacerbate this problem. This is a highly political issue, but it calls into question how to fund registries, what is the purpose of a registry, and who owns the data and rules to gain access. Another concern is the lack of a metric for survival despite its importance in planning and decision making.^7^ Incidence is needed to compute survival, but the latter is often ignored as adult registries de-emphasize it, instead focusing on risk prevention rather than treatment. Without detailed epidemiologic data collection by paying attention to critical demographic factors such as age, sex, and cancer type, profiling the cancer burden is difficult. Several factors contribute to this systemic problem, including lack of funding, difficulties in case finding, limited diagnostic capacity, lack of treatment facilities, low public awareness, and the general poor development of health information systems.^40^

At a service delivery level, health management information system development initiatives continue to take a strong hold in Africa and mandatory reporting of many disease entities by all health facilities is required by most African ministries of health.^41^ Some countries—although a small minority (ie, Uganda and Malawi)—in sub-Saharan Africa are now commendably leveraging these platforms to integrate palliative care indicators into the national health management information system.^42,43^ This is important not only for monitoring service performance and coverage but also for generating longitudinal data to inform large-scale quality evaluations and the development of area-based quality improvement plans to enhance efficiency in service delivery. These countries are, however, just two beacon sites.

At the child and family level, there is a dearth of evidence on best care models and outcomes in PPC.^22^ In Africa, patients with cancer can receive care at home, within communities, in outpatient settings, or in the inpatient ward. Data must therefore be captured across these care models. Although most countries have data at the health facility level, community-based health information systems remain largely underdeveloped; however, this is where the majority of patients are cared for and where data are most needed. Additionally, the care pathways experienced by children with different types of cancers have not been documented in the region. These pathways could inform the development of interventions geared toward promoting the detection of early signs, early referrals from all key points of health care systems, and enrollment into pediatric oncology programs to reduce late presentation rates, which have long been documented as a cause for poor prognosis.^44^

Moreover, the WHO consistently recommends person-centered health systems to optimize care outcomes. Now that progress is being made in configuring person-centered PPC and in developing person-centered outcome measures, regional efforts to promote the implementation of these measures in cancer care should be prioritized. The African Children's Palliative Care Outcome scale is a novel promising outcome tool that measures the physical, social, and psychologic well-being outcomes of the child and their family. In this way, integrated pediatric cancer care services will be more responsive to the needs of children and their families, as evidenced by such practices being associated with improved communication among patients, families, and health care providers, improved care satisfaction, and some health outcomes.^11,45^ Although evidence for the effectiveness of palliative care has been demonstrated in adult populations, prospective intervention-related data in pediatrics is scant. For example, a randomized prospective clinical trial by Temel et al^46^ demonstrated that early palliative care can improve the QoL and median survival time of patients with metastatic non–small-cell lung cancer, whereas some of the best pediatric-related data include studies such as the one conducted in Asia that demonstrated that home-based PPC in the last year of life reduced hospital admissions and medical costs and improved QoL.^12^ Notably, as stated earlier, a robust evidence base does not exist for children, especially in resource-limited settings.

## ACCESS TO ESSENTIAL MEDICINES

Lack of access to essential medicines largely contributes to the recognized inequity in the survival rates for patients with pediatric cancer and difficulties in treating symptom-related distress. Cure rates in developed countries exceed 80%, but remain very poor in developing countries, where up to 90% of cases occur.^47^ The WHO 2017 Global Cancer resolution, which includes childhood cancer in its cancer control mandate, highlights survival inequity as a major concern requiring attention,^48^ and as such, equitable access to essential medication should remain a priority. Measuring access to essential medicines remains a global challenge and a largely under-researched area and receives less attention when reporting on the Sustainable Development Goals.^49^ The situation is even more wanting when it comes to profiling access to pediatric chemotherapeutics.^50^ The International Society of Pediatric Oncology pointed out the urgent need for data on access to chemotherapeutics, with a strong recommendation that this should be tracked in the essential medicines list for children.^51^ The tracking of performance on availability can inform progress in interventions such as those of the Clinton Health Access Initiative and the American Cancer Society to increase access to cancer treatment in developing countries^52^ and promote health as a human right.

The combination of limited and poor resource management, both human and financial, results in inadequate treatment infrastructure that disproportionately affects children with cancer.^1^ Stefan et al reported the status of diagnostic and treatment facilities in Africa from 16 population-based cancer registries and showed that many centers lacked adequate diagnostic and treatment facilities, leading to the underdiagnosis of pediatric cancers. The study also highlighted that many childhood cancers had higher incidence rates on the continent than in developed countries.^40^

## HEALTH SYSTEM FINANCING

Generally, funding for palliative care is limited and few financial resources are available for PPC. Funding has been further affected by the 2008 global economic downturn, and the number of palliative care committed donors has been declining over time. Large donors who have left palliative care include the Diana, Princess of Wales Memorial Fund palliative care initiative and President's Emergency Plan for AIDS Relief.^53^ The situation has been further exacerbated by the economic consequences of the COVID-19 pandemic, pushing the already marginalized care approach to the peripheries of many international funding agendas. Given the evidence that palliative care improves many outcomes, including potential survival,^46^ it is important to further strengthen the evidence base for cost and cost-effectiveness^12^ and inform advocacy efforts toward the integration of palliative care into Universal Health Coverage (UHC).

## LEADERSHIP AND GOVERNANCE

States partly demonstrate their commitment to treating cancer by establishing national cancer control plans. These plans detail strategies to address the population burden of cancer through interventions to reduce its incidence, morbidity, and mortality and enhance the QoL of those at risk of or experiencing cancer. These plans outline the interventions required, how they will be implemented, who will be involved, the resources needed, and how they will be monitored and evaluated. In a global analysis on availability of cancer control plans, in the low-income cluster (n = 24), 22% had a national cancer control plan *and* a national communicable disease control plan, 67% had noncommunicable disease control plan alone, 7% had a national cancer control plan alone, and 4% others. In the low-middle–income cluster (n = 38), 53% had a national cancer control plan *and* a national communicable disease control plan, 39% had national communicable disease control plan alone, and 8% had a national cancer control plan alone.^54^ Almost all African countries fall within the group without national cancer control plans, resulting in cancer interventions for children and adults that are poorly planned and resourced. This adversely affects health promotion, cancer prevention, early diagnosis, treatment, rehabilitation, and palliative care. Additionally, only 26% of the plans assessed in low-income countries included PPC.^54^

## FORWARD-LOOKING PRIORITIZED AGENDA

Clearly, although some African countries have ongoing processes to deliver better palliative care for children and young adults with cancer, in the majority of sub-Saharan Africa, palliative care in pediatric oncology remains critically underdeveloped and neglected. To address the inequitable access to pediatric palliative oncology care, a multipronged agenda is essential. Following the WHO health system building blocks, this agenda includes the following:

- Health service delivery

Prioritize the development of person-centered PPC, combined with research initiatives to build an evidence base for the effectiveness of appropriate models of care.

- Health workforce

Opportunities for regional learning exist to share resources and identify avenues for training through established centers of excellence in different parts of Africa. Attempts should be made to leverage existing networks, such as the African Organization for Research and Training in Cancer, the International Society for Pediatric Oncology Africa, the Society for Neuro-Oncology Sub-Saharan Africa, the East African Center of Excellence in Oncology at the Uganda Cancer Institute, the African Cancer Institute, and the Africa Radiation Oncology Network.

Approximately nine countries have a national palliative care plan or program, and continuous development of education and training opportunities has been recorded in the region,^37^ with curricula integrating PPC. These should serve as an opportunity to strengthen pediatric cancer services by integrating PPC to deliver care that meets the needs of patients and their families.

- Health information systems

Additional effort is needed to support African countries to develop palliative care indicators within the context of pediatric cancer care.^55^ Countries should further be supported to integrate these indicators into large databases to track national-level performance and identify areas for improvement. It is also important to ensure that such data are captured across the disease trajectory, from diagnosis and treatment to remission, survivorship or end-of-life care, and bereavement.^56^

- Access to essential medicines

There is an acute need to collect data on the availability of essential medicines in the region to aid the tracking of performance in interventions geared toward scaling access to essential medicines. Moreso, it is urgent that the inequities in treatment access are addressed to improve the QoL and survival rates for children with cancers. There are regional efforts to increase access to cancer and symptom-related treatment, and access to affordable medicines is one of the interventions. Some initiatives include those by the American Cancer Society, the Clinton Access Initiative, and IBM, which are scaling up access to more affordable generic cancer medicines.

- Health system financing

Financing for cancer control and treatment should be integral to regional response strategies and should be integrated into all control plans. PPC plans should be integrated into all national cancer plans to facilitate PPC funding.

- Leadership and governance

For health systems to deliver a person-centered approach, PPC should be integral to regional and national cancer control plans.^29^ As the region advances the UHC coverage agenda, integrated pediatric cancer palliative care should be central to efforts to address inequity in access to quality care for this vulnerable segment of the population.^57^ A minimum package for palliative care under UHC for Africa has been proposed,^58^ and regional partners should advocate for its inclusion in their national health policies and guidelines.^59^

We realize that this agenda is partly premised on local, intracontinental, and international training and on advocacy partnerships to mitigate the negative impact of woefully inadequate provision of pediatric oncology palliative care services. Foremost, regional co-operative blocks, such as the East African Community, the Southern African Development Community, and the West Africa Development Union, need to embrace the strategies proposed by the Union for International Cancer Control and the African Palliative Care Association, to embrace basic national cancer control policies for each country in their respective subregion. Second, the regional blocks should include cancer and pain or palliative medicines within their national essential drug list and leverage policy to train professional nurse prescribers for pain or palliative medicines, particularly for children and young adults with cancer. This effort mandates the establishment and strengthening of local training programs intended to promote the integration of PPC in mainstream oncology services and the extension of pediatric palliative oncology care to the primary care level, thus improving the referral and follow-up of children and young adults with palliative care needs during cancer care.

In conclusion, health systems for pediatric oncology services in sub-Saharan Africa are largely characterized by a lack of well-resourced national cancer control plans and inadequate financing, resulting in poor or no cancer diagnostic and treatment and training infrastructure, limited or no human resource capacity, and poor or no access to palliative care. Additionally, the level of PPC development in the region remains inadequate and continues to lag behind that for adult palliative care.^14^ Given the evidence that children with cancer face multidimensional symptoms and concerns across the disease trajectory, countries must prioritize funding for and integration of PPC within pediatric oncology.

## Data Availability

No additional data available.

## References

[1] Ward ZJ Yeh JM Bhakta N, et al: Global childhood cancer survival estimates and priority-setting: A simulation-based analysis. Lancet Oncol 20:972-983, 2019 31129029 10.1016/S1470-2045(19)30273-6

[2] Buckle GC Collins JP Sumba PO, et al: Factors influencing time to diagnosis and initiation of treatment of endemic Burkitt lymphoma among children in Uganda and western Kenya: A cross-sectional survey. Infect Agent Cancer 8:36, 2013 24079452 10.1186/1750-9378-8-36 PMC3849966

[3] International Agency for Research on Cancer: World Cancer Report 2014, Lyon France, IARC

[4] Moore MA Shin HR Curado MP, et al: Establishment of an Asian Cancer Registry Network—Problems and perspectives. Asian Pac J Cancer Prev 9:815-832, 2008 19256782

[5] Stefan DC: Patterns of distribution of childhood cancer in Africa. J Trop Pediatr 61:165-173, 2015 25724211 10.1093/tropej/fmv005

[6] Nsondé Malanda J Nkoua Mbon JB Bambara AT, et al: Twelve years of working of Brazzaville cancer registry. Bull Cancer 100:135-139, 2013 23406573 10.1684/bdc.2013.1701

[7] Bhakta N Force LM Allemani C, et al: Childhood cancer burden: A review of global estimates. Lancet Oncol 20:e42-e53, 2019 30614477 10.1016/S1470-2045(18)30761-7

[8] Bray F Ferlay J Soerjomataram I, et al: Global cancer statistics 2018: GLOBOCAN estimates of incidence and mortality worldwide for 36 cancers in 185 countries. CA Cancer J Clin 68:394-424, 2018 30207593 10.3322/caac.21492

[9] Worldwide Palliative Care Alliance, WHO: Global Atlas of Palliative Care at the End of Life. London, UK, Worldwide Palliative Care Alliance, 2014

[10] World Health Assembly: WHO Strengthening of Palliative Care as a Component of Comprehensive Care Throughout the Life Course, 2014, 2014. http://apps.who.int/gb/ebwha/pdf_files/WHA67/A67_R19-en.pdf

[11] Wolfe J Orellana L Cook EF, et al: Improving the care of children with advanced cancer by using an electronic patient-reported feedback intervention: Results from the PediQUEST randomized controlled trial. J Clin Oncol 32:1119-1126, 2014 24616307 10.1200/JCO.2013.51.5981 PMC3970170

[12] Chong PH De Castro Molina JA Teo K, et al: Paediatric palliative care improves patient outcomes and reduces healthcare costs: Evaluation of a home-based program. BMC Palliat Care 17:11, 2018 29298714 10.1186/s12904-017-0267-z PMC5751774

[13] Connor SR Downing J Marston J: Estimating the global need for palliative care for children: A cross-sectional analysis. J Pain Symptom Manage 53:171-177, 2017 27765706 10.1016/j.jpainsymman.2016.08.020

[14] Rhee J Luyirika E Namisango E, et al: APCA Atlas for Palliative Care, 2017

[15] Berwick DM: The total customer relationship in health care: Broadening the bandwidth. Jt Comm J Qual improv 23:245-250, 1997 9179716 10.1016/s1070-3241(16)30314-5

[16] WHO: Monitoring the Building Blocks of Health Systems: A Handbook of Indicators and Their Measurement Strategies. Geneva, Switzerland, WHO, 2010

[17] Namisango E Bristowe K Allsop MJ, et al: Symptoms and concerns among children and young people with life-limiting and life-threatening conditions: A systematic review highlighting meaningful health outcomes. Patient 12:15-55, 2019 30361884 10.1007/s40271-018-0333-5

[18] Ruland CM Hamilton GA Schjødt-Osmo B: The complexity of symptoms and problems experienced in children with cancer: A review of the literature. J Pain Symptom Manage 37:403-418, 2009 18694633 10.1016/j.jpainsymman.2008.03.009

[19] Israels T Kambugu J Kouya F, et al: Clinical trials to improve childhood cancer care and survival in sub-Saharan Africa. Nat Rev Clin Oncol 10:599-604, 2013 23897077 10.1038/nrclinonc.2013.137

[20] WHO: Definition of Palliative Care 2013. http://www.who.int/cancer/palliative/definition/en/

[21] Weaver MS Yao AJ Renner LA, et al: The prioritisation of paediatrics and palliative care in cancer control plans in Africa. Br J Cancer 112:1845-1856, 2015 26042935 10.1038/bjc.2015.158 PMC4580392

[22] Harding R Sherr L Albertyn R: The Status of Paediatric Palliative Care in Sub-Saharan Africa—An Appraisal. London, UK, The Diana, Princess of Wales Memorial Fund, King's College London, 2010

[23] Friedel M Aujoulat I Dubois AC, et al: Instruments to measure outcomes in pediatric palliative care: A systematic review. Pediatrics 143:e20182379, 2019 30530504 10.1542/peds.2018-2379

[24] Donabedian A: The quality of care. How can it be assessed? JAMA 260:1743-1748, 1988 3045356 10.1001/jama.260.12.1743

[25] Harding R Selman L Agupio G, et al: Validation of a core outcome measure for palliative care in Africa: The APCA African Palliative Outcome Scale. Health Qual Life Outcomes 8:10, 2010 20100332 10.1186/1477-7525-8-10 PMC2825183

[26] Namisango E Bristowe K Murtagh FE, et al: Towards person-centred quality care for children with life-limiting and life-threatening illness: Self-reported symptoms, concerns and priority outcomes from a multi-country qualitative study. Palliat Med 34:319-335, 2020 32081084 10.1177/0269216319900137

[27] Namisango E Luyirika E Allsop MJ, et al: Characteristics and symptom prevalance in children and young people with cancer: A meta-analysis, in Kenya National Palliative Care Conference 2018. Nairobi, Kenya, Kenya Hospices and Palliative Care Associations, 2018, pp 9

[28] Kingham TP Alatise OI Vanderpuye V, et al: Treatment of cancer in sub-Saharan Africa. Lancet Oncol 14:e158-e167, 2013 23561747 10.1016/S1470-2045(12)70472-2

[29] WHO: WHO Global Strategy on People-Centred and Integrated Health Services: Interim Report. Geneva, Switzerland, World Health Organization, 2015

[30] Kreicbergs UC Lannen P Onelov E, et al: Parental grief after losing a child to cancer: Impact of professional and social support on long-term outcomes. J Clin Oncol 25:3307-3312, 2007 17664479 10.1200/JCO.2006.10.0743

[31] Worldwide Hospice and Palliative Care Alliance: Global Atlas of Palliative Care (ed 2). London, UK, Worldwide Hospice and Palliative Care Alliance, 2020

[32] Harif M: Addressing inequalities in oncology care for African children. EBioMedicine 62:103140, 2020 33249383 10.1016/j.ebiom.2020.103140 PMC7701321

[33] Stefan DC: Childhood cancer in Africa: An overview of resources. J Pediatr Hematol Oncol 37:104-108, 2015 24487917 10.1097/MPH.0000000000000111

[34] Bretscher M Rummans T Sloan J, et al: Quality of life in hospice patients. A pilot study. Psychosomatics 40:309-313, 1999 10402876 10.1016/S0033-3182(99)71224-7

[35] Friedrichsdorf SJ Remke S Symalla B, et al: Developing a pain and palliative care programme at a US Children's Hospital. Int J Palliat Nurs 13:534-542, 2007 18073700 10.12968/ijpn.2007.13.11.27588

[36] Luyirika EB Namisango E Garanganga E, et al: Best practices in developing a national palliative care policy in resource limited settings: Lessons from five African countries. Ecancermedicalscience 10:652, 2016 27563347 10.3332/ecancer.2016.652 PMC4970625

[37] Rawlinson F Gwyther L Kiyange F, et al: The current situation in education and training of health-care professionals across Africa to optimise the delivery of palliative care for cancer patients. Ecancermedicalscience 8:492, 2014 25624873 10.3332/ecancer.2014.492 PMC4303614

[38] Harding R Selman L Powell RA, et al: Research into palliative care in sub-Saharan Africa. Lancet Oncol 14:e183-e188, 2013 23561750 10.1016/S1470-2045(12)70396-0

[39] Gakunga R Parkin DM: Cancer registries in Africa 2014: A survey of operational features and uses in cancer control planning. Int J Cancer 137:2045-2052, 2015 26135162 10.1002/ijc.29668

[40] Stefan C Bray F Ferlay J, et al: Cancer of childhood in sub-Saharan Africa. Ecancermedicalscience 11:755, 2017 28900468 10.3332/ecancer.2017.755 PMC5574662

[41] Kiberu VM Matovu JK Makumbi F, et al: Strengthening district-based health reporting through the district health management information software system: The Ugandan experience. BMC Med Inform Decis Mak 14:40, 2014 24886567 10.1186/1472-6947-14-40 PMC4030005

[42] Palliative Care Association of Uganda: Civil Society Report on Palliative Care in Uganda. Kampala, Uganda, Palliative Care Association of Uganda, 2019

[43] Palliative Care Association of Malawi: Ministry of Health Integrates Palliative Care Indicators in the District Health Information System, 2018. https://www.palliativecareassociationofmalawi.org

[44] Makerere University College of Health Sciences (MakCHS), Uganda Cancer Institute (UCI): Training Needs Assessment for the East African Centre of Excellence in Oncology. Kampala, Uganda, Makerere University, 2019

[45] Etkind SN Daveson BA Kwok W, et al: Capture, transfer, and feedback of patient-centered outcomes data in palliative care populations: Does it make a difference? A systematic review. J Pain Symptom Manage 49:611-624, 2015 25135657 10.1016/j.jpainsymman.2014.07.010

[46] Temel JS Greer JA El-Jawahri A, et al: Effects of early integrated palliative care in patients with lung and GI cancer: A randomized clinical trial. J Clin Oncol 35:834-841, 2017 28029308 10.1200/JCO.2016.70.5046 PMC5455686

[47] Rodriguez-Galindo C Friedrich P Alcasabas P, et al: Toward the cure of all children with cancer through collaborative efforts: Pediatric oncology as a global challenge. J Clin Oncol 33:3065-3073, 2015 26304881 10.1200/JCO.2014.60.6376 PMC4979198

[48] WHO: Cancer Prevention and Control in the Context of an Integrated Approach. 2017. https://apps.who.int/iris/handle/10665/275676

[49] Wirtz VJ Hogerzeil HV Gray AL, et al: Essential medicines for universal health coverage. Lancet 389:403-476, 2017 27832874 10.1016/S0140-6736(16)31599-9 PMC7159295

[50] Barr R Robertson J: Access to cytotoxic medicines by children with cancer: A focus on low and middle income countries. Pediatr Blood Cancer 63:287-291, 2016 26375626 10.1002/pbc.25722

[51] Denburg A Arora B Arora RS, et al: Access to essential medicines for children with cancer: A joint SIOP-CCI position statement. Lancet Oncol 18:20-22, 2017 28049570 10.1016/S1470-2045(16)30652-0

[52] American Society: American Cancer Society and Clinton Health Access Initiative Announce Collaborations with Pfizer and Cipla to Increase Access to Lifesaving Cancer Treatment in Africa, 2017. http://pressroom.cancer.org/2017-06-20-American-Cancer-Society-and-Clinton-Health-Access-Initiative-Announce-Collaborations-with-Pfizer-and-Cipla-to-Increase-Access-to-Lifesaving-Cancer-Treatment-in-Africa

[53] Callaway M Foley KM De Lima L, et al: Funding for palliative care programs in developing countries. J Pain Symptom Manage 33:509-513, 2007 17482039 10.1016/j.jpainsymman.2007.02.003

[54] Romero Y Trapani D Johnson S, et al: National cancer control plans: A global analysis. Lancet Oncol 19:e546-e55, 2018 30268693 10.1016/S1470-2045(18)30681-8

[55] Baker JN Levine DR Hinds PS, et al: Research priorities in pediatric palliative care. J Pediatr 167:467-470.e3, 2015 26028284 10.1016/j.jpeds.2015.05.002 PMC4516589

[56] Allsop M Kabukye J Powell R, et al: Routine Data and Minimum Datasets for Palliative Cancer Care in Sub-Saharan Africa: Their Role, Barriers and Facilitators, in Silbermann M (eds): Palliative Care for Chronic Cancer Patients in the Community, Basel, Switzerland, Springer, Cham, 2021

[57] Knaul FM Farmer PE Krakauer EL, et al: Alleviating the access abyss in palliative care and pain relief-an imperative of universal health coverage: The Lancet commission report. Lancet 391:1391-1454, 2018 29032993 10.1016/S0140-6736(17)32513-8

[58] Knaul FM: Integrating palliative care into health systems is essential to achieve Universal Health Coverage. Lancet Oncol 19:e566-e7, 2018 30344078 10.1016/S1470-2045(18)30600-4

[59] African Palliative Care Association: Essential Package for Universal Health Coverage, 2019. https://palprac.org/wp-content/uploads/2019/11/Palliative-care-in-Universal-Health-Care-package-.pdf

